# What Happened Pre- and during COVID-19 in South Korea? Comparing Physical Activity, Sleep Time, and Body Weight Status

**DOI:** 10.3390/ijerph18115863

**Published:** 2021-05-29

**Authors:** Jeong-Hui Park, Eunhye Yoo, Youngdeok Kim, Jung-Min Lee

**Affiliations:** 1 Department of Physical Education, Kyung Hee University (Global Campus), 1732 Deokyoungdaero, Giheung-gu, Yongin-si 17014, Korea; jeonghee@khu.ac.kr; 2 Department of Physical Education, Seoul National University, 1 Gwanakro, Gwanak-gu, Seoul 08826, Korea; yeh04@snu.ac.kr; 3 Department of Kinesiology and Health Sciences, Virginia Commonwealth University, 907 Floyd Ave, Richmond, VA 23284, USA; kimy13@vcu.edu; 4 Sports Science Research Center, Kyung Hee University (Global Campus), 1732 Deokyoungdaero, Giheung-gu, Yongin-si 17014, Korea

**Keywords:** COVID-19, social distancing, physical activity, sleep time, body weight status

## Abstract

The purpose of the current study is to investigate the changes in physical activity (PA), sleep time (ST), and body weight (BW) Pre- and during COVID-19 in South Korea, and specifically, PA data were obtained during COVID-19 at three-time points based on the multilevel social distancing policies. All data were surveyed by questionnaires online and offline, and participants were required to fill in the monthly average of daily step counts were recorded an application on participants’ smartphone devices from Pre-COVID-19 (2019 year) and during COVID-19 (2020 year). Participants were 834 adults (males: 54.4%, female: 45.6%) and all statistical analyses were summarized by SPSS 25.0 program. The monthly average of daily step counts was 6747.09 during Pre-COVID-19, but the PA during COVID-19 was 5812.11 daily step counts per month. Also, there were significant pairwise differences between average PA Pre-COVID-19 and each level of social distancing (*p* < 0.001). After COVID-19, the participants who slept less than 7 h decreased by 3.6%, while those who slept more than 9 h increased by that much. As a result of BW, 269 participants responded their BW changed during COVID-19, and 199 of them reported they gained BW during COVID-19 (74.0%). Although self-reported questionnaires may have led to an under-or over-estimation of ST and BW, the present study found that the environment in which the COVID-19 is prevalent had adverse relationships on PA, ST, and BW. Therefore, it is important to identify strategies to motivate individuals for remaining physically active and getting adequate sleep while maintaining social distancing due to the presence of the COVID-19 global pandemic.

## 1. Introduction

Regular participation in physical activity (PA) provides not only physiological benefits by lowering the risk of mortality from cardiovascular and metabolic diseases [1,2,3,4], but also immunological benefits mediated by attenuation of inflammation, increased circulation of immunoglobulin, and anti-inflammatory cytokines due to enhanced macrophage reactions [5,6]. The World Health Organization has proposed PA guidelines and encouraged people to engage in a minimum of 150 min per week of moderate-intensity aerobic PA 75 min per week of vigorous-intensity aerobic PA, or equivalent combination [7]. Furthermore, there is growing evidence showing a reciprocal association between PA and body weight (BW) and sleep health [8,9]. The recent findings suggest that promoting PA and limiting prolonged bouts of sedentary time is beneficially associated with BW [10], and specifically, it is well-documented that regular and appropriate PA has associated with better sleep health [11]. Also, a greater chance to have sufficient sleep time (ST), and conversely, getting enough ST plays a critical role in promoting PA by regulating immune cells and repairing heart and blood vessels during sleep [12,13,14]. Many previous studies demonstrated that ST was associated with various health problems such as cardiovascular diseases, diabetes, obesity, and mental health [15,16,17]. Thus, the National Sleep Foundation (NSF) has established age-specific guidelines for optimal sleep duration, in which adults aged from 18 to 64 years are recommended to have an average sleep between 7 to 9 h per day [18].

Nevertheless, there are multiple factors influencing compliance with recommendations of the public health (i.e., PA and ST) [19,20], which include personal characteristics, social environment, and natural environments such as seasonal characteristics and weather changes [21,22]. A critical potential natural environmental factor that has recently attracted the most attention is the highly contagious epidemic (i.e., Severe Acute Respiratory Syndrome: SARS, Middle East Respiratory Syndrome: MERS, H1N1 Influenza). For instance, one study investigated the health-seeking behaviors of the general public pre- and post-SARS and reported that most people had increased the frequency of hygienic behaviors for health (i.e., using health services, mask-wearing, and frequent hand-washing) [23]. However, despite increased interest in their healthy diet and weight control, the time of participation in PA per week has decreased since SARS (pre: 59.2%, post: 58.9%) and will gradually decrease in the future (OR = 0.963) [23]. In addition, many countries have used limited behavior (i.e., social distancing, isolation, and quarantine) as important aspects of preventing the spread of infectious diseases [24,25]. Some studies have shown that national policies such as social distance control to prevent infectious diseases are related to public physical health. For example, studies have shown that weight also increases as sedentary behavior increased [26,27]. There are growing constraints on public health due to environmental and social factors, compared with the past. However, the most significant health inhibitor at present is the global pandemic called COVID-19.

The global COVID-19 pandemic, which first broke out in Wuhan, China, in December 2019, is an acute respiratory infection and has spread at an unprecedented rate compared to previous epidemics. With the rapidly increasing number of confirmed cases worldwide, the World Health Organization (WHO) declared COVID-19 to be a ‘pandemic’ on 11 March 2020. To avoid infection, people drastically cut down on outdoor activity and each country has announced various measures to prevent the spread of COVID-19 based on their governance [28,29,30,31], and the Korean government also has specially adopted different levels of social distancing, not only to prevent infection and the spread of COVID-19 but also to preserve the daily routine and economic activities of the citizens and prepare for the long haul.

Although the current study has some limitations such as an under-or overestimation of participants’ PA, ST, and BW through self-reported questionnaires, the primary aim of this study is to investigate the changes in health-related behaviors (i.e., PA and ST) and outcomes (i.e., BW) Pre-COVID-19 (From January 2019 to December 2019) and during COVID-19 (From January 2020 to December 2020). Additionally, the secondary aim is to explore the relationship between the social distancing policy and behavioral outcomes (i.e., PA, ST, and BW) during the outbreak of highly contagious COVID-19 (From March 2020 to December 2020) and identify differences in the outcomes among gender and participants’ different residential areas (i.e., urban, suburban, and rural).

## 2. Methods

### 2.1. Study Participants

The current study used a convenience sampling method to recruit participants and distribute online questionnaires through university-based e-mail and offline questionnaires to students, faculty, and staff in various regions’ universities (urban, suburban, and rural). Also, their families, friends, and relatives could participate in the present study. The eligibility criteria for responding to questionnaires in this study are as follows: (a) adults over 18 years old who have never been infected with COVID-19, (b) adults who were using iPhone smartphone at least once from January 2019 to October 2020, and (c) adults who lived in South Korea from January 2019 to October 2020. 

To minimize COVID-19 infections, the questionnaires were distributed and collected when there were the fewest confirmed cases and when the social distancing was at the lowest level (October 2020). Participants were also asked to complete online or offline surveys that they could choose to do on their own, and they were able to respond to the questions at their own pace, at any time, and anywhere they could access the questionnaire for this survey. All participants responded with a free choice between online or offline survey, and about 80% of 1000 potential respondents returned completed the survey for the study (*n* = 849). Among the survey participants, 142 participants responded through the online survey, and the rest of the participants responded through the offline questionnaire of the survey (*n* = 692). However, some participants who did not never report the monthly average of daily step counts (*n* = 157) and misstates the monthly average of daily step counts (*n* = 21) were excluded, so only 618 participants recorded the monthly average of daily step counts across 22 consecutive months (from January 2019 to October 2020). Also, among the 849 participants, 345 participants who did not respond to ST both Pre- and during COVID-19 were excluded, so a total of 504 participants recorded ST. 504 participants responded to the average ST Pre-COVID-19 (From January 2019 to December 2019), and 502 adults recorded the average ST during COVID-19 (From January 2020 to October 2020). On the other hand, all 834 adults responded to this study’s survey on changes in BW Pre- and during COVID-19, so there are no missing values. The Institutional Review Board approved the present study of Kyung Hee University (KHGIRB-21-099). 

### 2.2. Levels of Social Distancing

The levels of social distancing defined by the Korea Disease Control and Prevention Agency (28 June 2020) are as follows: Level 1 (i.e., June, July, and October 2020) is “distancing in daily life,” which is adopted when there are less than 50 daily confirmed cases within the community. Gatherings, meetings, events, and the use of facilities by large numbers of people (both public and private) are permitted only when complying with disinfection rules. Level 2 (i.e., March, April, and August 2020) is adopted when there are 50–100 daily confirmed cases for two weeks. In this scenario, it is strongly recommended to curtail gatherings, meetings, and events and shut down many people’s facilities (both public and private). Level 2.5 (i.e., September 2020) is adopted when there are at least 100–200 confirmed daily cases during the past two weeks. Gatherings, meetings, and events with 50 persons or more if indoors and 100 persons or more if outdoors are prohibited. However, due to financial hardships stemming from the economic slowdown, stores are permitted to open from 9 a.m. to 5 p.m., only for takeout and delivery. As such, Korean society has been gradually making efforts to prevent the spread of COVID-19 at the governmental level. 

### 2.3. Measures

#### 2.3.1. Device-Based Physical Activity 

Data for this study were obtained from participants’ iPhone smartphones (Apple Inc., Cupertino, CA, USA), and participants had to use iPhone smartphones at least once from January 2019 to October 2020. Also, participants were asked to report the monthly average of daily step counts from the health app from their smartphone in the questionnaire (Pre-COVID-19: *n* = 576, level 1: *n* = 597, level 2: *n* = 587, and level 2.5: *n* = 587). Apple’s health application automatically measures the users’ daily steps, and users easily track their daily, monthly, and yearly step counts. The Apple health application has shown that Mean Absolute Percentage Errors (MAPEs) values are small compared to ActiGraph wGTX+ and it revealed high validity for the step counts measured in various conditions (i.e., different speeds and wearing locations) among other applications (i.e., Garmin Vivofit 2, Samsung’s Health, Moves, Runtastic Pedometer, Accupedo, and Pacer) [32]. Modern smartphones contain a variety of sensors such as the global positioning system and built-in accelerometer application (e.g., Apple’s Health app and Samsung’s S Health app) [33], and the applications utilize a three-axis gyroscope, accelerometer, and M9 motion coprocessor, so they can easily capture and access PA (i.e., daily steps) [34]. Furthermore, several studies have demonstrated that smartphones with built-in accelerometers are a powerful device to examine for large-scale population and their health, revealing the basic patterns of PA [35,36]. Specifically, the monthly average of daily step counts measured by smartphones has provided accurate and reliable PA estimates in both laboratory and free-living settings [33,37]. Therefore, although participants may not always carry their smartphone, given the PA data tracked over 22 months, this study can analyze participants’ PA patterns between Pre- and during COVID-19.

#### 2.3.2. Self-Reported Sleep Time

Participants were asked for the average daily sleep time pre- and during COVID-19. Participants responded as continuous variables to the question ‘How many hours did you sleep a day on average Pre-COVID-19 (2019 year)?’ and ‘How many hours do you sleep a day during the COVID-19 (2020 year)?’. The National Sleep Foundation (NSF) has recommended for adults aged from 18 to 64 year to have an average sleep between 7 to 9 h per day [18], so the present study classified into three categories: less than 7 h, from 7 to 9 h, and more than 9 h.

#### 2.3.3. Self-Reported Body Weight Changes 

Participants answered ‘yes’ or ‘no’ to a question about BW changes: ‘Has your body weight changed Pre- and during COVID-19?’. If the participant responded with ‘yes’, one of the following questions should be filled in the changed BW: (a) If you gained weight, how much did you gain?, (b) If you lost weight, how much did you lose?’. The current study categorized the participants’ BW changes reported as continuous variables into three categories based on percentile: from 1 to 2 kg (25th percentile), from 3 to 5 kg (50th percentile), and more than 5 kg (75th percentile).

#### 2.3.4. Other Study Covariates

Demographic characteristics, including age (years), gender, location (urban, suburban, and rural), education (less than high school, high school diploma, some college, college graduate, and graduate degree), annual household income (<1000 thousand KRW, 1000–<2000 thousand KRW, 2000–<3000 thousand KRW, and ≥3000 thousand KRW), occupation (office workers, administrators, managers, and professionals, skilled agricultural and fishery workers, students, and jobless), and married (married or living as married and single) were obtained. Also, anthropometric information, including height (cm) and weight (kg), was obtained, and body mass index (BMI) was calculated by dividing the weight (kg) by the square of the height (m^2^). Thus, COVID-19 infection was confirmed as ‘yes’ or ‘no’.

### 2.4. Statistical Analysis

All statistical analyses were summarized by SPSS 25.0 (SPSS Inc., Chicago, IL, USA), and descriptive statistics examined the participants’ personal information for demographics and anthropometrics. The monthly average of daily step counts was analyzed using the paired *t*-test and compared with each month between Pre-COVID-19 (i.e., 2019 year) and during COVID-19 (i.e., 2020 year). The p-value was utilized to demonstrate whether the significant difference between participants’ PA (i.e., daily step counts) Pre- and during COVID-19. Mean and standard deviations (SD) were calculated for PA, ST, and BW changes, and *p* *<* 0.05 was set as the limit for statistical significance.

To examine the changes in PA by social distancing policy, this study classified Pre-COVID-19 (2019 year), level 1, level 2, and level 2.5 for the 2020 year, and a one-way ANOVA was performed. To be specific, the association with gender, location, and three different levels of social distancing for PA were evaluated by two-way ANOVA (gender × level of social distancing, location × level of social distancing) and three-way mixed-model ANOVA (gender × location × level of social distancing).

## 3. Results

The demographic and anthropometric characteristics of the participants are presented in Table 1. The participants (54.4% males, 45.6% females) were on average 23.7 ± 6.0 years and at a level of education above college (100%). Most of the participants were students (*n* = 742, 89.0%), so the monthly average income accounted for the highest percentage of <1000 thousand KRW (USD, <915) (*n* = 394, 47.2%). Also, participants were mostly single (*n* = 774, 92.8%), and all participants had never been infected with COVID-19 (*n* = 834, 100%).

### 3.1. Average Daily Step Counts per Month and Level of Social Distancing in COVID-19

The comparison of average step counts across gender, location, and level of social distancing (i.e., level 1, level 2, and level 2.5) are presented in Table 2. The overall average PA was 6747.09 steps per month Pre-COVID-19, but the PA during COVID-19 was 5812.11 steps per month and PA decreased as the level of social distancing increased. To be specific, Post-hoc analyses indicated that there were significant differences in average PA between Pre-COVID-19 and each level of social distancing (*p* < 0.001), however, there was no significant difference between level 2 and level 2.5 (*p* = 1.000). Furthermore, PA across gender and social distancing levels have no significant differences (*p* > 0.05), but it showed a similar pattern of decreasing PA in both males and females until level 2 (males: from 7002.45 to 5892.16, females: from 6500.44 to 5347.04). Likewise, interaction with PA and location × social distancing level has tended to decrease significantly during COVID-19 than Pre-COVID-19 (*p* < 0.05). Although there were no significant differences in PA across gender × location × level of social distancing (*p* > 0.05), both males and females in each location became less participated in PA during COVID-19, which decreased as the level of social distancing was raised except for PA in rural from level 2 (males: 6063.09, females: 4769.10) to level 2.5 (males: 6167.73, females: 5799.15).

### 3.2. Average Daily Step Counts per Month and Pre- and during COVID-19

**Figure 1:**
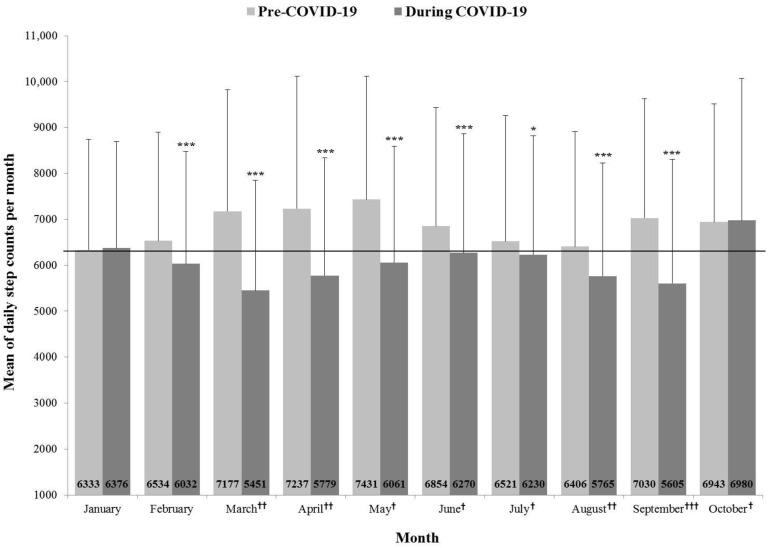
Mean of daily step counts per month before and during COVID-19. *** *p* < 0.001, * *p* < 0.05; †: Level 1 of social distancing in COVID-19, ††: Level 2 of social distancing in COVID-19, †††: Level 2.5 of social distancing in COVID-19.

This study compared the average monthly step counts from January 2019 to October 2020 by matching the same month in different years (2019 year and 2020 year) (Figure 1). In the rest of the months except January and October, there were significant differences in average monthly step counts between pre-and during COVID-19 (January, February, March, April, May, June, August, and September: *p* < 0.001, July: *p* < 0.05). Also, since the social distancing during the COVID-19 was implemented (March 2020), the participants’ PA decreased dramatically compared to Pre-COVID-19, which was lowered in order of level 1 (May, June, July, and October 2020), level 2 (March, April, and August 2020) and level 2.5 (September 2020).

### 3.3. Average Sleep Time and Pre- and during COVID-19

**Figure 2:**
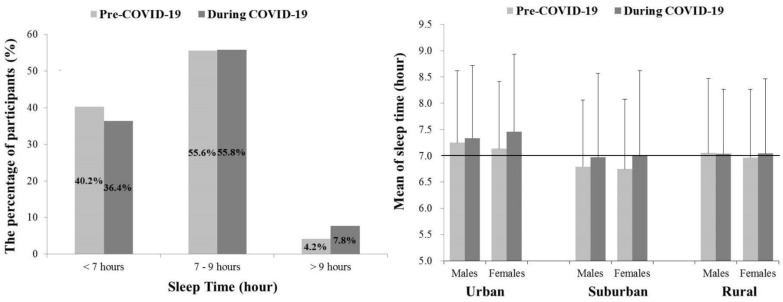
Mean of sleep time before and during COVID-19. Urban: includes a capital and metropolis city in South Korea, Suburban: includes a nearby city that surrounds the capital city, Rural: includes the rest of the cities except urban and suburban.

A total of 503 participants responded to the average daily ST Pre-COVID-19 (2019 year) and during COVID-19 (2020 year) (Figure 2). Pre-COVID-19, 55.6% of participants slept from 7 to 9 h, followed by 40.2% of participants who slept less than 7 h. This pattern was similar during the COVID-19, but 36.4% of the participants slept less than 7 h (3.6% down), and participants who slept more than 9 h increased (Pre-COVID-19: 4.2%, During COVID-19: 7.8%). In addition, although there were no significant differences in ST across gender × location during COVID-19, compared to Pre-COVID-19 (*p* > 0.05), differences in ST between Pre- and during COVID-19 showed an increase in urban and suburban, compared to rural.

### 3.4. Body Weight and Pre- and during COVID-19

**Figure 3:**
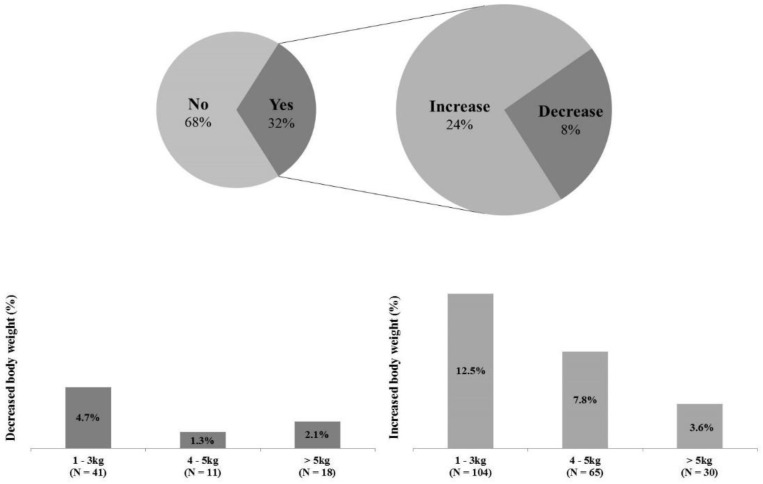
Changed in body weight before and during COVID-19. Among a total of 834 participants, 565 participants (68.0%) responded “maintenance” to the body weight questions, and 269 participants (32.0%) answered “changes”. Of the participants who answered ‘changes’, 199 participants (24.0%) filled in the increased weight, and 70 participants (8.0%) recorded the decreased weight.

All participants answered questions about changes in BW Pre- and during the COVID-19 (Figure 3). 565 participants (68.0%) responded “maintenance” to a question about whether there was any change in their BW during COVID-19, and 269 participants (32.0%) answered “changes”. Among the participants who answered ‘changes’, 199 participants (24.0%) filled in the increased weight, and 70 participants (8.0%) recorded the decreased weight.

## 4. Discussion

The possibility of new infectious diseases is increasing due to frequent international exchanges (e.g., trade and travel) between countries and climate change [38,39,40]. Therefore, the international communities have emphasized cooperation in the outbreak of infectious diseases and the need to strengthen its response to the global epidemic of new infectious diseases such as the COVID-19 crisis [41,42,43]. As part of that, the purpose of the study is to examine how the COVID-19 affected our lives. The present study investigated the changes in PA, ST, and BW Pre- and during the COVID-19 and compared the changes across gender, three different locations (i.e., urban, suburban, and rural), and levels of social distancing (i.e., Level 1, Level 2, and Level 2.5) implemented by adaptive governance in South Korea.

### 4.1. Average Daily Step Counts per Month and Level of Social Distancing in COVID-19

The environment in which the COVID-19 has prevalent a significant relationship with participants’ daily step counts. Compared to Pre-COVID-19, average daily step counts were significantly reduced during the COVID-19 (Pre-COVID-19: 6747.09 steps and during COVID-19: 5812.11 steps) consistent with the results of previous studies [44,45] and this current study demonstrated that social distancing also negatively impacted participants’ daily steps. As the level of social distancing increased, the individuals seemed to be trying to minimize movement. Specifically, average daily step counts in Level 1 and Level 2 showed significant differences (*p* < 0.001) although there was no significant difference between daily steps in Level 2 and Level 2.5 (*p* < 0.05). These results are likely to increase participants’ anxiety about the infection of COVID-19 by getting ratcheted up from Level 1 to Level 2, so they made an effort to minimize their activities since Level 2. The daily step counts in urban and suburban also tended to decrease as the level elevated, but participants in rural increased when the level of social distancing changed from Level 2 to Level 2.5 (Level 2: 5358.23 ± 2471.12 and Level 2.5: 5966.95 ± 2858.90). The reason may be that the rural adults were typically less wary of their activities because of its low population density and relatively fewer confirmed cases than other locations (i.e., urban and suburban). It is hard to know whether rural adults’ daily steps have increased while maintaining social distance, but the results showed the possibility of increasing steps despite the high level of social distancing. Therefore, it should be considered to increase their activities while maintaining social distancing and complying with the preventive policy about infectious diseases.

### 4.2. Average Daily Step Counts per Month and Pre- and during COVID-19

The present study demonstrated that the COVID-19 has a significantly adverse relationship between average daily step counts Pre- and during the COVID-19. According to previous studies, people’s step counts tend to be low in winter (i.e., December, January, February) and summer (i.e., June, July, and August) [46,47,48], but typically high in spring (i.e., March, April, May) and autumn (i.e., September, October, and November) [49,50]. However, during the COVID-19, the average step counts per month began to decrease gradually in January and indicated the lowest step counts in March and September during the COVID-19 that was Level 2 of social distancing. In addition, summer (June, July, and August) during COVID-19 showed a relative increase in steps than other months It may have increased their activities for participants because the level of social distancing was lower than in March and April. On the other hand, daily step counts in October increased significantly during the COVID-19 because it was likely to aggregate the lowest number of confirmed cases during the COVID-19 (*n* = 15), and there was a holiday (October 9) and Korea’s traditional holidays (from September 30 to October 4). Therefore, these seasonal and social issues and COVID-19 can be described as closely related. 

### 4.3. Average Sleep Time and Pre- and during COVID-19

The National Sleep Foundation (NSF) reported a sleep duration recommendation that adults over 18 years should have 7–9 h of sleep [18]. In this study, there was little change in the percentage of participants who slept 7–9 h Pre- and during COVID-19 (Pre-COVID-19: 55.6%, During COVID-19: 55.8%). However, the percentage of participants who slept less than 7 h decreased (Pre-COVID-19: 40.2%, During COVID-19: 36.4%), and the percentage of participants who slept more than 9 h increased (Pre-COVID-19: 4.2%, During COVID-19: 7.8%). This result is likely to be due to the changing social environment during COVID-19. Most stores and cafes had to close early (from 9 a.m. to 5 p.m.), and universities and workplaces had to comply with non-face-to-face for preventing infection of the virus, so these situations that had to stay at home longer may be contributed to the increase of participants’ ST. Furthermore, participants have saved time spent on commuting and had flexible schedules during COVID-19, which may be allowed to extend sleep duration. A similar finding has reported in previous studies observed people’s ST during the COVID-19 [18,51], but we need to be careful of excessive sleep for more than 9 h because people with excessive ST (more than 9 h) showed the lower quality of life and productivity in school and workplace [52,53]. 

### 4.4. Body Weight and Pre- and during COVID-19

In the present study, participants who maintained their BW Pre- and during COVID-19 accounted for higher percentages than participants who experienced BW changes (maintenance: 68%, changes: 32%). However, even if there were fewer participants with changes in BW, 32% of those who experienced weight changes answered that they had gained BW during COVID-19 than Pre-COVID-19 (increase: 24%, decrease: 8%). It is difficult to determine the cause of weight loss because the present study did not investigate the cause of weight change, but participants who reported weight gain were able to speculate the cause based on step counts and ST results of the current study, and several epidemiological studies. The increase in BW may be a necessary result because the participants’ step counts during COVID-19 have been significantly lower than Pre-COVID-19 and the ST has increased based on the present study. In addition, some studies reported that people’s eating habits have changed (i.e., delivered foods intake, increasing snack intake, and irregular eating) since COVID-19 Pandemic [54,55], which might be contributed to gain BW. Even people’s dependence on alcohol has increased significantly to relieve stress and depression caused by COVID-19 [56,57,58], which would promote weight gain.

### 4.5. Limitations and Future Perspectives

The current study comes with some limitations. First of all, we measured participants’ PA, ST, and BW through self-reported questionnaires because the face-to-face measurement was limited due to prevent COVID-19 infections, so it may have led to an under-or overestimation of PA, ST, and BW. To minimize the problem, future studies need accurate measurements of these variables. Also, we have only quantitative assessments of ST and BW in this study and did not investigate qualitative assessments of the quality of sleep and the causes of BW. Therefore, concerning the qualitative assessment of sleep and BW results, it is difficult to discuss it in the study. In addition, it was difficult to accurately measure physical activity because the number of steps was measured around the participants who owned the iPhone. Therefore, it is necessary to use valid physical activity assessment tools. Finally, the majority of participants in the study were young adults (18–40 years) and this study was conducted on only Koreans. If further studies are conducted in more diverse age groups and countries repeatedly, it may have a more positive effect on generalization.

## 5. Conclusions

Although self-reported questionnaires may have led to an under-or over-estimation of ST and BW, the present study found that the environment in which the COVID-19 is prevalent had adverse relationships on PA, ST, and BW. Therefore, it is important to identify strategies to motivate individuals for remaining physically active and getting adequate sleep while maintaining social distancing due to the presence of the COVID-19 global pandemic. In particular, there were the association with daily step counts and mortality, cardiovascular disease (CVD) risk, and type 2 diabetes, and participating in additional 1000 steps per day was associated with lower risk of all-cause mortality, and lower risk of CVD morbidity or mortality [59,60]. Therefore, PA promotion strategies to be implemented in the future should be simple but enjoyable, and anyone can participate, and specific and feasible PA and ST guidelines for each level of social distancing should be developed.

## Figures and Tables

**Table 1:** Characteristics of Participants (*n* = 834).

Variable		All Participants	
		No. (%)	Mean ± SD
**Age (year)**			23.7 ± 6.0
Gender	Male	454 (54.4)	
	Female	380 (45.6)	
Anthropometrics	Height (cm)		170.0 ± 8.9
	Weight (kg)		64.8 ± 13.5
	BMI (kg·m^-2^)		22.3 ± 3.7
Location	Urban	212 (25.4)	
	Suburban	427 (51.2)	
	Rural	177 (21.2)	
Education	Less than high school	0 (0.0)	
	High school diploma	0 (0.0)	
	Some college	712 (85.4)	
	College graduate	79 (9.5)	
	Graduate degree	43 (5.2)	
Annual Household Income	<1000 thousand KRW (USD < 915)	394 (47.2)	
	1000–2000 thousand KRW (USD 915–1830)	43 (5.2)	
	2000–3000 thousand KRW (USD 1830–2745)	34 (4.1)	
	≥3000 thousand KRW (USD ≥ 2745)	48 (5.8)	
Occupation	Office workers	23 (2.8)	
	Administrators, managers, and professionals	19 (2.3)	
	Skilled agricultural and fishery workers	44 (5.3)	
	Students	742 (89.0)	
	Jobless (e.g., housewife)	5 (0.6)	
Married	Married or living as married	58 (7.0)	
	Single	774 (92.8)	
Infection of COVID-19	Yes	0 (0.0)	
	No	834 (100.0)	

SD: standard deviation, BMI: Body Mass Index; Urban: includes a capital and metropolis city in South Korea, Suburban: includes a nearby city that surrounds the capital city, Rural: includes the rest of the cities except urban and suburban; Total number of persons responded 816 at the location, and 2.2% missing from the table (did not answer), Total number of persons responded 519 at income, and 37.8% missing from the table (did not answer), Total number of persons responded 833 at occupation, and 0.1% missing from the table (did not answer), Total number of persons responded 832 at married, and 0.2% missing from the table (did not answer), 1 USD = 1092.80 KRW (Korean Won, 15 December 2020).

**Table 2:** Average daily step counts divided by gender, location, and gender × location in three different levels of social distancing (Mean ± SD).

Variable			Pre-COVID-19 (*n* = 576)	during COVID-19		
				Level 1 (*n* = 597)	Level 2 (*n* = 587)	Level 2.5 (*n* = 587)
**Overall**			**6747.09 ± 1987.55**	6208.77 ± 2320.85 ***	5616.35 ± 2181.45 ***	5611.19 ± 2702.18 ***
Gender	Male		7002.45 ± 1992.47	6545.95 ± 2379.93	5892.16 ± 2199.26	5659.44 ± 2553.51
	Female		6500.44 ± 1944.64	5877.19 ± 2215.57	5347.04 ± 2133.38	5564.39 ± 2842.47
Location	Urban		6986.71 ± 1926.39	6236.50 ± 2269.87	5462.51 ± 1973.94	5474.28 ± 2706.81
	Suburban		6778.90 ± 1933.82	6241.32 ± 2120.55	5786.31 ± 2121.34	5497.52 ± 2552.63
	Rural		6380.80 ± 2162.28	6037.84 ± 2740.61	5358.23 ± 2471.12	5966.95 ± 2858.90
Gender × Location	Urban	Male	7280.85 ± 1659.71	6639.77 ± 2099.91	5790.21 ± 1936.97	5701.71 ± 2442.82
		Female	6791.79 ± 2071.11	5963.01 ± 2350.71	5251.58 ± 1979.71	5325.27 ± 2870.63
	Suburban	Male	7000.71 ± 2000.49	6395.19 ± 2162.82	5875.33 ± 2134.69	5488.27 ± 2361.61
		Female	6495.83 ± 1813.43	6043.16 ± 2055.61	5671.76 ± 2106.34	5509.27 ± 2785.26
	Rural	Male	6738.55 ± 2300.28	6921.85 ± 3215.30	6063.09 ± 2681.75	6167.73 ± 3186.09
		Female	6100.82 ± 2020.65	5309.84 ± 2027.60	4769.10 ± 2126.18	5799.15 ± 2566.35

SD: standard deviation; *** *p* < 0.001; Urban: includes a capital and metropolis city in South Korea, Suburban: includes a nearby city that surrounds the capital city, Rural: includes the rest of the cities except urban and suburban; Level 1: There are less than 50 daily confirmed cases within the community, and gatherings, meetings, events, and the use of facilities by large numbers of people (both public and private) are permitted, Level 2: There are 50–100 daily confirmed cases for two weeks and gatherings, meetings, and events reduced and facilities used by many people (both public and private) shut down, Level 2.5: There are at least 100–200 confirmed daily cases during the past two weeks and gatherings, meetings, and events with 50 persons or more if indoors, and 100 persons or more if outdoors, are prohibited, in particular, stores are permitted to open from 9 a.m. to 5 p.m.

## Data Availability

The datasets used and/or analyzed during the current study are available from the corresponding author on reasonable request.
